# Intense pulsed light as a multi-target intervention paradigm for refractory ocular surface diseases: a review

**DOI:** 10.1186/s40662-026-00510-3

**Published:** 2026-09-30

**Authors:** Jiayi Yan, Wei Chen, Jinyang Li

**Affiliations:** National Clinical Research Center for Ocular Diseases, Eye Hospital, Wenzhou Medical University, Wenzhou, 325027 China

**Keywords:** Intense pulsed light, Meibomian gland dysfunction, Blepharitis, Chalazion, Ocular rosacea, Allergic conjunctivitis, Photobiomodulation, Multi-target therapy

## Abstract

Ocular surface diseases (OSDs) are a heterogeneous group of disorders characterized by overlapping inflammatory, vascular, microbial, neuroimmune, and meibomian gland abnormalities that contribute to the onset and persistence of ocular surface symptoms and signs. Conventional symptom-directed therapies do not adequately alleviate refractory meibomian gland dysfunction, Demodex-associated blepharitis, ocular rosacea, recurrent chalazion, and allergic conjunctivitis. Recent evidence suggests that these conditions may share convergent pathological features, including chronic inflammation, abnormal lid-margin vascularity, and microbial or parasitic imbalance. Intense pulsed light (IPL), which was initially used for vascular and inflammatory disorders in dermatology, has emerged as a potential physical therapy that acts through several proposed mechanisms in patients with selected refractory OSDs, including photothermal liquefaction of inspissated meibum, coagulation of abnormal vessels, reduction of Demodex and microbial burden, modulation of inflammatory responses, and possible effects on neuroimmune signaling; however, these mechanisms remain incompletely established. Case series and clinical studies have reported improvements in ocular symptoms, tear film stability, meibomian gland function and drainage, lid-margin inflammation, and chalazion or lesion resolution, particularly when IPL is combined with meibomian gland expression or other adjunctive therapies. However, treatment responses vary across disease phenotypes and standardized treatment parameters, long-term safety data, and high-quality randomized controlled trials remain limited. Anatomical factors, disease severity, glandular structure, and inflammatory status may also influence therapeutic outcomes. Overall, IPL therapy represents a promising but evolving treatment strategy for refractory OSDs. This review summarizes the current knowledge regarding the proposed mechanisms, clinical applications, treatment protocols, limitations, and future directions and provides updated perspectives for the rational use of IPL in OSD management.

**Supplementary Information:**

The online version contains supplementary material available at https://doi.org/10.1186/s40662-026-00510-3.

## Background

Since the 1990 s, intense pulsed light (IPL) based on selective photothermolysis has been used in dermatology to treat vascular and pigmentary lesions [1–3]. IPL is effective in treating rosacea, benign vascular lesions, and pigmented lesions because the target chromophores absorb specific wavelengths of light and convert them into thermal energy.

In 2002, Dr. Toyos reported a notable clinical observation that bridged dermatology and ophthalmology: patients undergoing facial IPL for rosacea experienced improvement in concomitant refractory dry eye [4]. This serendipitous observation suggested a clinically relevant overlap between periocular skin disease and ocular surface inflammation. As an anatomical extension of the facial skin, the eyelid shares common pathological targets with the skin, including abnormal vascular proliferation, chronic inflammation, and microbial imbalance [5, 6]. This shared “eyelid-facial” pathological mechanism provides a rationale for repurposing IPL, which had originally been designed to occlude abnormal vasculature and remove pigmented targets, as a multi-target modality for refractory ocular surface diseases (OSDs). Accordingly, IPL protocols have gradually been optimized and standardized for treating evaporative dry eye, and its indications have extended to include blepharitis and recurrent chalazia [3, 4, 7–9].

OSD is a broad group of diseases involving the structure and function of the ocular surface, including blepharitis, chalazion, and meibomian gland dysfunction (MGD) [10–12]. It is a significant public health problem with a global prevalence of 5%–50% [13–16]. Dryness and discomfort affect daily activities and can cause psychological and social discomfort in severe cases [13–16].

In this heterogeneous group, several refractory OSDs, including MGD, Demodex-associated blepharitis, ocular rosacea, and chalazia, are unified pathophysiologically by the self-perpetuating vicious cycle of chronic inflammation, abnormal vascular proliferation, glandular dysfunction, and microbial imbalance [10, 11, 16–18]. These pathological processes may contribute to a secondary cascade at the ocular surface: inflammation breaches the epithelial barrier and disrupts meibomian gland function to elicit an abnormal tear film [17, 19]. This unstable hyperosmolar tear film traps inflammatory mediators and allergens, further aggravating the primary inflammation [20]. This may reinforce a self-perpetuating cycle of inflammation → barrier disruption → tear film abnormality → intensified inflammation [17, 20].

Symptomatic therapies, such as artificial tears, topical anti-inflammatory agents, and antibiotics, target manifestations in the secondary cascade, including tear film instability and inflammation, but not the drivers of this cascade, namely aberrant vasculature supplying inflammatory mediators, physical obstruction of the meibomian glands, and microbiological imbalance maintaining inflammation [21, 22]. This may partly explain why some patients, especially those with refractory diseases, show incomplete or short-term responses to pharmacological treatments.

By selectively targeting the underlying disease processes through the photothermal occlusion of pathological vessels, liquefaction of the inspissated meibum, reduction of Demodex burden, and photobiomodulation of cellular metabolism, IPL may provide a mechanistically distinct modality that targets several components of this vicious cycle. However, the relative contribution of each pathway remains unclear.

Nevertheless, some concerns have been expressed about its universal application, including a lack of standardized treatment parameters, few high-quality randomized controlled trials, and incomplete understanding of its mechanisms, which may be related to neuromodulation and the immune microenvironment. However, when considering the clinical manifestations, the leading pathological driver of such varied OSDs converges on a standard, self-perpetuating vicious circle: “inflammation–vascular abnormalities–microbiome imbalance–glandular dysfunction.” This review provides a narrative overview of IPL therapy for refractory OSDs, focusing on proposed mechanisms, clinical applications, treatment protocols, limitations, and future directions. This review aims to clarify a possible common pathophysiological pathway through which IPL may act multimodally and to evaluate its potential clinical role in patients with poor responses to conventional drug therapy or limited adherence to long-term home-based treatment. Thus, this review provides a theoretical and clinical framework for the rational, indication-specific use of IPL while emphasizing that the strength of evidence differs substantially across disease categories.

## Main text

## Mechanism of IPL therapy for OSDs

**Fig. 1:**
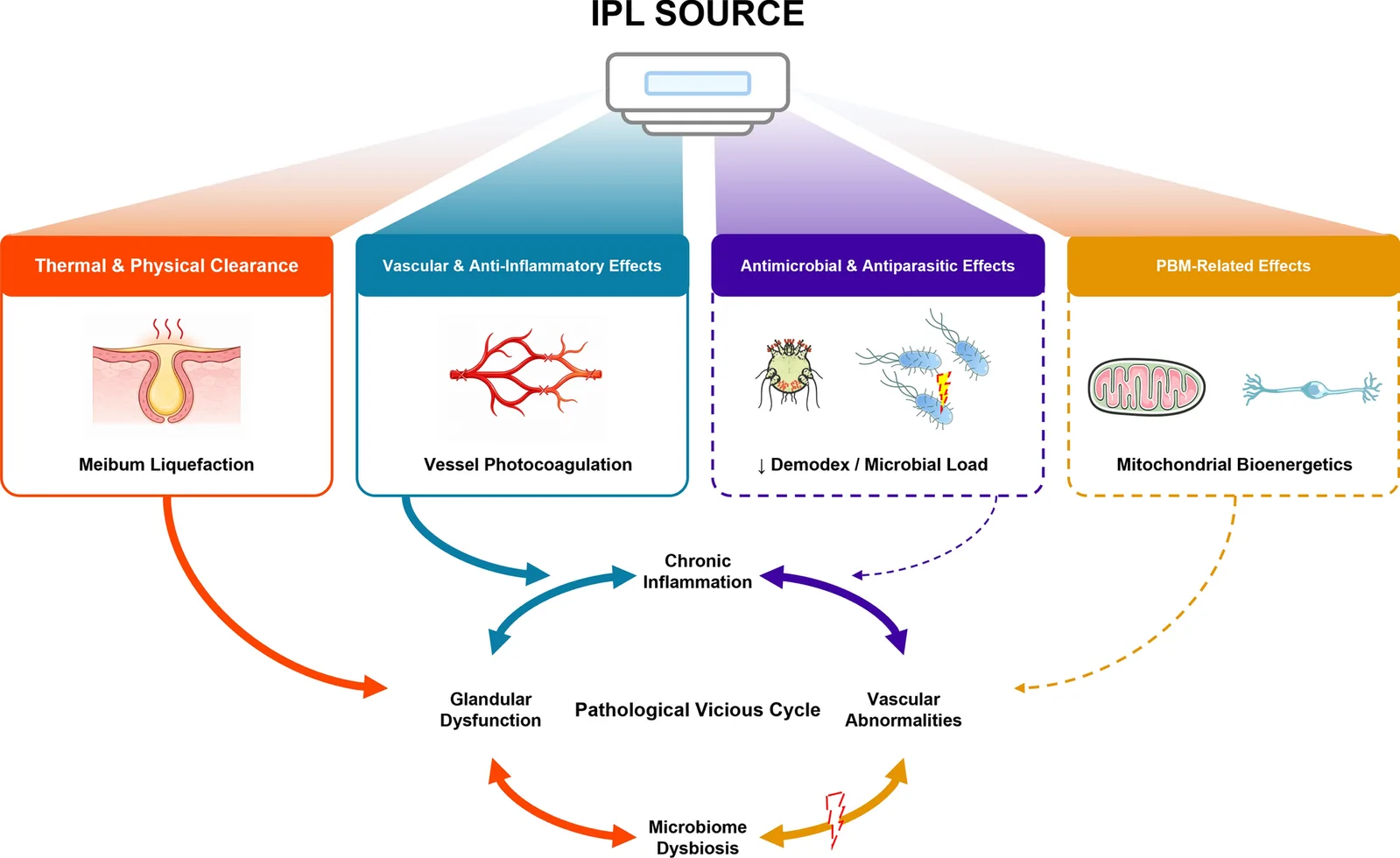
Proposed multi-target mechanisms of intense pulsed light (IPL) therapy in ocular surface disease. IPL may modulate the interconnected cycle of chronic inflammation, vascular abnormalities, microbiome dysbiosis, and glandular dysfunction through four partially overlapping pathways: thermal and physical clearance with liquefaction of the inspissated meibum and improved glandular or ductal patency, photocoagulation of abnormal or telangiectatic vessels with downstream inflammatory modulation, reduction of Demodex and microbial load, and possible photobiomodulation-related effects on mitochondrial bioenergetics, cellular repair, and neurosensory signaling. Solid outlines and arrows indicate relatively established mechanisms, whereas dashed outlines, dashed arrows, and lightning symbols indicate proposed or emerging mechanisms that require further validation

Its therapeutic effects may be understood within a multi-target treatment framework aimed at several overlapping pathological features of OSDs. As depicted in Fig. 1, IPL may modulate the components of the pathological cycle involving inflammation, vascular abnormalities, microbiome dysbiosis, and MGD through four overlapping mechanisms.

### Thermal effects and physical drainage

IPL therapy is based on the principle of selective photothermolysis, whereby the light of specific wavelengths is preferentially absorbed by chromophores in target tissues and converted into heat. In MGD, the resulting photothermal effect helps in reducing the viscosity of the inspissated meibum and improves its fluidity within the gland ducts. When combined with subsequent mechanical gland expression, IPL facilitates meibum evacuation through a “thermal liquefaction–mechanical drainage” effect, thereby improving ductal obstruction and meibomian gland secretion [1, 23–28]. Clinical studies support this synergistic effect, demonstrating substantial improvements in the meibomian gland yielding secretion score with a mean difference of +13.69 at 1 month post-treatment [26]. Overall, the photothermal effect of IPL provides a plausible mechanism for improving gland obstruction in MGD. However, the therapeutic response may vary according to disease severity, glandular structural damage, device parameters, and whether meibomian gland expression (MGX) was performed.

### Anti-inflammatory effects

IPL is absorbed by hemoglobin within pathologically dilated periorbital capillaries; it generates heat and induces microthrombi and vascular occlusion. Subsequent structural interruptions may reduce the delivery of inflammatory mediators to the ocular surface. Clinically, this manifests as a reduction in hyperemia and edema of the eyelid margin, bulbar conjunctiva, and limbus after treatment [23, 29–33].

At the molecular level, IPL is associated with changes in tear inflammatory cytokines. Studies have demonstrated marked decreases in interleukin (IL)-6, tumour necrosis factor-α (TNF-α), C-X-C motif ligand 1, and interferon-γ (all *P* < 0.001), accompanied by a significant reduction in matrix metalloproteinase-9 (MMP-9) levels—with positivity decreasing from 97% to 69% (*P* = 0.026) and significantly reduced signal intensity (*P* = 0.011)—and a concomitant increase in tissue inhibitor of metalloproteinases-1 (*P* < 0.001) [23, 34–36]. These changes correlate with clinical improvements: tear film breakup time (TBUT) prolongation is associated with decreased TNF-α (R = −0.814, *P* = 0.014), while improvements in meibomian gland secretion score and ocular surface disease index (OSDI) correlate with reduced levels of multiple pro-inflammatory cytokines (all *P* < 0.05) [23, 34, 36–38]. Many of these cytokines are algogenic and may contribute to the sensitization of corneal nerve endings, evoking pain and foreign-body sensations [20]. These cytokine changes may attenuate nociceptive signaling and neurosensory symptoms; however, direct evidence that IPL reverses neurosensitization remains limited [30, 38]. Together with its possible effects on neurosensory pathways, IPL may reduce neuropathic symptoms, partly by decreasing inflammatory nociceptive input; however, whether it also promotes neural repair requires further study [38, 39]. In summary, IPL may exert anti-inflammatory effects. At the vascular level, it physically occludes the pathological vessels that supply inflammatory mediators; at the molecular level, it downregulates pro-inflammatory cytokines and upregulates protective factors. This combined action reduces both the objective signs of ocular surface inflammation and subjective neurosensory symptoms.

### Antibacterial and antiparasitic effects

Demodex infestation and increased bacterial load are primary contributors to MGD and related ocular surface inflammation. Chromophores in mite exoskeletons and internal pigments may absorb IPL, leading to a localized temperature increase and potential thermal injury. In vitro, standard IPL parameters kill live Demodex mites, and clinical studies have reported a significant post-treatment decrease in mite counts on the eyelid margin [29, 40–42].

Concomitantly, IPL reduces the bacterial load at the eyelid margin by disrupting the bacterial cell walls. Demodex often harbors bacteria and releases inflammatory substances, including lipases. IPL may reduce mite burden and bacterial load, thereby decreasing local inflammatory stimuli. These effects may contribute to reduced ocular surface inflammation and lower levels of inflammatory mediators, including MMP-9 [23, 35, 43]. In summary, IPL may reduce Demodex and bacterial burden at the eyelid margin, thereby decreasing local inflammatory stimuli and complementing conventional antimicrobial therapies.

### Photobiomodulation and tissue repair

The red and near-infrared regions of the IPL spectrum penetrate the tissue and are absorbed by cytochrome c oxidase in the mitochondrial membrane. Non-thermally, the increased mitochondrial membrane potential may improve the efficiency of the electron transport chain, stimulate adenosine triphosphate (ATP) synthesis, and influence cellular metabolic, proliferative, and repair-related pathways [44, 45]. This has been suggested by cellular-level findings using flavoprotein fluorescence technology: after IPL treatment, the flavoprotein fluorescence significantly decreased from 13,800 ± 3200 to 10,200 ± 2800 a.u. (*P* < 0.001), which is consistent with increased mitochondrial metabolic activity and reduced oxidative stress [39].

One possible explanation for the frequent clinical observation that symptomatic relief precedes objective structural recovery is that IPL-related bioenergetic and inflammatory changes may contribute to early relief from neurosensory irritation. In contrast, measurable glandular structural recovery may require a longer period of tissue remodeling [38]. IPL improves microstructural parameters, such as meibomian gland acinar unit density increasing from 78.5 ± 15.2 to 96.3 ± 18.7 glands/mm^2^ (*P* < 0.01), further suggesting increased glandular cell activity [21]. IPL has been reported to enhance fibroblast proliferation and modulation of matrix metalloproteinase-1 and transforming growth factor-β1 (TGF-β1) expression, potentially supporting extracellular matrix remodeling and providing a more favorable microenvironment for glandular functional recovery [46–50]. In intractable OSDs, these photobiomodulatory effects may be associated with neurosensory and immune-related changes, suggesting a possible shift from symptom control to the modulation of neuroimmune and tissue repair pathways. However, this remains an emerging hypothesis [38, 39].

In summary, IPL- or low-level light therapy (LLLT)-related photobiomodulatory effects have been proposed to modulate cellular energy metabolism, inflammatory signaling, and neurosensory symptoms. These proposed phototherapeutic mechanisms are summarized in Supplementary Table 1. However, direct evidence of neuroimmune restoration, neural repair, or tissue regeneration in OSD remains limited. Therefore, these effects should be interpreted as emerging mechanisms rather than as established clinical outcomes.

Each phototherapeutic modality uniquely emphasizes the underlying mechanisms. For instance, LLLT may provide greater short-term improvements in patient-reported symptoms and basal tear secretion, whereas IPL primarily acts on vascular and inflammatory mechanisms [51]. Thus, the combination of IPL and LLLT may allow broader symptom and tear film improvements through complementary mechanisms [38, 39, 52, 53]. A randomized controlled trial further confirmed that this combination therapy showed greater improvement than sham treatment in OSDI and TBUT [54]. However, the exact cellular and molecular signaling pathways through which IPL functions remain elusive and need to be elucidated [45]. Based on these mechanisms and shared pathophysiological features, IPL has evolved from treatment for evaporative dry eye to a potential adjunctive therapy for selected refractory OSDs. Four major disease categories treated with IPL are discussed below according to their dominant pathological drivers and available clinical evidence. Allergic conjunctivitis is discussed separately as an emerging adjunctive application because the current clinical evidence remains limited.

1. Lipid-abnormality type: MGD, characterized by ductal obstruction and altered meibum secretion [55, 56].
2. Immune-allergic type: allergic conjunctivitis, driven primarily by immunoglobulin E (IgE)-mediated hypersensitivity and immune dysregulation [57–61].
3. Infection–inflammation mixed type: blepharitis associated with Demodex infestation and disruption of the lid-margin microenvironment [62, 63].
4. Vasculo-neurogenic dysregulation type: ocular rosacea, defined by chronic facial and periocular vascular dysregulation [6, 64].
5. Chronic granulomatous type: chalazion presenting as sterile lipogranulomatous inflammation secondary to meibomian-gland obstruction [7, 65, 66].

## Standardization and individualized setting of IPL treatment parameters

The clinical effectiveness of IPL therapy for MGD and related OSDs depends on accurate parameter settings. Based on its multi-target mode of action, the current academic consensus is that parameter settings should be dynamic and personalized according to skin phototype, lesion characteristics, and treatment area.

### Core physical parameters

Several clinical trials have established the following main parameters of IPL therapy.

- Wavelength selection: the IPL device emits polychromatic light in the range of 515–1200 nm; therefore, filters are needed to select the desired wavelength. Most studies chose the 590 nm or 560 nm filter to treat telangiectasia along the eyelid margin [24]. Hemoglobin absorbs well in this wavelength range, enabling the selective photothermal closure of abnormal vessels. This event decreases the amount of inflammatory mediators released from the source, thereby exerting an anti-inflammatory effect.
- Energy density: energy density settings are critical parameters for ensuring effectiveness and safety and are thus usually determined according to the Fitzpatrick skin phototype classification. For low-risk lighter skin types I–III, the common energy level is 12–16 J/cm^2^ [24, 67]. For darker skin types or pigmentation risk types IV–V, the energy level is generally lowered to 9–11 J/cm^2^ to avoid damaging the epidermis [68, 69].
- Pulse mode: clinically, the tripulse mode is typically applied to ensure sufficient thermal accumulation within the target tissue while preventing burns on the epidermis [24]. Millisecond-scale interpulse delays allow the epidermis to dissipate heat and permit thermal accumulation in deeper meibomian glands to reach the phase transition temperatures that liquefy the pathological meibum.

### Upper eyelid treatment

The application of eye protection technology provides critical safety assurance for direct IPL exposure of the eyelids. With complete corneal and scleral coverage using a Jaeger opaque metal eye shield, direct irradiation of the upper eyelid skin at an energy density of 10 J/cm^2^ has been reported to be safe and effective. By employing the same eye protection technology, the clinical value of simultaneous IPL application to the upper and lower eyelids was further validated in Asian populations [24, 70]. This approach may extend treatment coverage to the upper eyelid glands, enabling functional intervention over the entire eyelid gland system and thus improving the overall therapeutic effect significantly [71].

Clinically, this intervention for the upper eyelid is significant because it expands the treatment range and improves treatment outcomes. Several studies have shown that morphological changes in the upper eyelid glands have a greater impact on IPL outcomes as demonstrated by the significantly higher scores for meibomian gland loss in the upper eyelid of patients in the non-responsive group. In addition, the negative prognostic indicator “sequential extreme thinning” is more common in the upper eyelids. These findings suggest that the involvement of the upper eyelid gland may affect IPL outcomes [71].

### Treatment protocol and course design

The therapeutic effects of IPL exhibit a significant cumulative effect.

- Treatment frequency: standard treatment involves three to four basic treatment sessions. For severe or refractory MGD, increasing the number of treatments may offer longer-lasting improvements in tear film stability (e.g., 8 treatments at 3-week intervals) [67–69, 71, 72].
- Treatment interval: usually, treatment sessions are spaced 2–4 weeks apart to provide continuous suppression of inflammatory responses while allowing adequate recovery time between sessions [24, 72].
- Efficacy prediction and individualized treatment: the effectiveness of IPL therapy varies among individuals, and baseline characteristics predict the treatment response. Patients with severe glandular structural damage, such as high meibomian gland loss scores and sequential extreme thinning, respond poorly to IPL therapy, even when the eyelid margin signs are mild. Meibomian gland photography is critical for predicting the prognosis of IPL, and clinical practice should integrate morphology and individualized treatment choices [71].

### Combined adjuvant therapy: IPL-MGX and LLLT

Although simple IPL irradiation liquefies meibum through photothermal effects, its transient thermal effect does not induce permanent patency of the glands. Therefore, immediate manual MGX after IPL treatment is highly recommended in clinical practice. Thermal conduction from IPL denatures and liquefies the occlusion, whereas mechanical expression extrudes accumulated meibum. Therefore, this combination is commonly used in clinical studies and clinical practice to treat MGD-related dry eye [52, 72, 73].

LLLT supplementation of IPL utilizes the photobiomodulatory effects of red or near-infrared light. It may enhance cellular metabolism and anti-inflammatory responses with possible effects on tissue repair-related pathways. Evidence suggests that combined regimens of IPL and LLLT have greater potential than monotherapies to improve subjective symptom scores and prolong initial TBUT, thus opening new perspectives for optimizing future treatment strategies [74–76].

## Application of IPL in various types of refractory OSDs

To summarize the representative clinical evidence for IPL therapy in refractory OSDs, we organized relevant studies across four major disease categories: MGD, blepharitis, chalazion, and ocular rosacea. As this is a narrative review rather than a systematic review, representative studies were selected from clinical trials, observational studies, case series, and reviews using search terms such as “intense pulsed light,” “IPL,” “ocular surface disease,” “dry eye,” “meibomian gland dysfunction,” “blepharitis,” “chalazion,” “ocular rosacea,” “allergic conjunctivitis,” and “Demodex.” Priority was given to studies that reported IPL parameters, clinical outcomes, safety considerations, and disease-specific applications. Because the evidence for allergic conjunctivitis remains limited, relevant studies on this condition are summarized as an emerging adjunctive application rather than as a major disease category. Supplementary Table 1 presents detailed study characteristics, including study design, patient populations, IPL parameters, and clinical outcomes.

### MGD

#### Disease overview and epidemiological characteristics

MGD is a chronic diffuse ocular disease characterized by obstruction of the meibomian gland ducts and abnormalities in the composition or amount of meibum secretion [10]. This is the leading cause of evaporative dry eye. The primary pathology of the disease involves overproduction of keratin in the ducts and changes in the chemical composition of the meibum, making it thicker and more difficult to remove. Recently, novel studies have led to the understanding that acinar renewal abnormalities, rather than ductal obstruction, are involved in MGD pathogenesis [77]. The resulting deficiency in the tear film lipid layer not only accelerates tear evaporation but also increases the risk of dry eye syndrome. This also initiates a vicious cycle of hyperosmolarity and chronic inflammation [56]. In certain Asian regions, the prevalence can reach 60%–70% [55, 78]. MGD also involves several other causative factors, including glandular dysfunction and varying hormone secretion levels [55]. MGD has recently been linked to allergic conjunctivitis in pediatric populations, in which pathogenic immune-mediated inflammation drives the severity of the disease at an early stage [9, 79, 80].

#### Treatment limitations and IPL evidence in MGD

Standard treatments for MGD include eyelid hygiene, warm compression, gland massage, artificial tears, and topical anti-inflammatory or antibiotic therapy [56, 79]. However, these approaches may be burdensome and do not adequately address glandular obstruction, abnormal lid-margin vascularity, or chronic inflammation in refractory cases [54, 56, 79, 81]. Current studies suggest that IPL can improve tear film stability, meibomian secretion quality, lipid layer thickness, inflammatory cytokine profiles, and patient-reported symptoms, especially when combined with MGX [25, 27, 34, 35, 37, 54, 67–69, 76, 82–86]. IPL has been prospectively evaluated against topical cyclosporine A and diquafosol sodium in patients with moderate-to-severe dry eye, and the results suggest that IPL may improve several tear film- and symptom-related outcomes over short-term follow-up [87]. Pediatric studies have also reported potential improvements in symptoms and meibomian gland morphology, although the evidence remains limited [88, 91]. Among the ocular surface conditions reviewed, MGD-related evaporative dry eye has the strongest evidence base for IPL, supported by randomized controlled trials and meta-analyses [14, 26, 31, 55, 68–70, 90]. Nevertheless, further studies are needed to optimize treatment parameters, validate long-term efficacy, and establish phenotype-driven protocols [14, 56, 71, 88].

### Blepharitis

#### Disease overview and epidemiology

Blepharitis is a chronic inflammatory disease of the eyelid margins. Clinically, the disease manifests as anterior and posterior blepharitis [11].

The main pathogenesis of this disease is Demodex mite infestation. Mites occlude the orifices of the gland ducts and act as bacterial vectors. They also trigger delayed hypersensitivity reactions, causing chronic inflammation of the ocular surface [62, 89, 90]. This condition is prevalent in ophthalmology, with prevalence rates rising sharply with age [91]. The worldwide prevalence of demodectic blepharitis is estimated at 41%–70%; mite detection is almost 100% in individuals aged over 70 years [90]. In addition to eye itching and dryness, disease-induced redness and swelling of the eyelids, with typical sleeve-like secretions and cylindrical crusts, place a high social and psychological burden on patients [89, 91, 92].

#### Treatment limitations and IPL evidence in Demodex-associated blepharitis

Conventional treatments for Demodex-associated blepharitis include lid hygiene, topical acaricidal agents, antimicrobial agents, and anti-inflammatory treatment [62, 90, 92]. However, long-term treatment can be irritating or burdensome, and recurrence is common in some patients [90–92]. Based on the general mechanisms discussed earlier, IPL has been explored as an adjunct option, particularly in patients with Demodex-associated or refractory blepharitis. Published studies suggest that IPL may reduce Demodex mite burden, lid-margin inflammation, hyperemia, and tear film instability in selected patients with Demodex-associated or refractory blepharitis, especially when combined with topical ivermectin or other light-based therapies [34, 41, 61, 62, 91–95]. However, the current evidence remains less robust than that for MGD-related evaporative dry eye, and further controlled studies are needed to determine the optimal treatment protocols and long-term outcomes [62, 63, 91].

### Ocular rosacea

#### Pathogenesis and epidemiology

Ocular rosacea is a complex inflammatory condition that affects the periorbital and ocular surface tissues [96, 97]. This condition results from the hyperactivity of the immune system, which triggers inflammatory and oxidative stress responses and disturbs ocular surface homeostasis. However, ocular rosacea remains underrecognized in clinical practice. According to a review published in 2025, ocular manifestations are found in up to 44.3% of patients with cutaneous rosacea [97]. However, ocular symptoms are diagnosed in only 10.3% of the cases, indicating a wide diagnostic gap between the two conditions [98]. This implies that ocular problems in many patients remain undiagnosed, and that earlier recognition may be important. The pathogenesis of ocular rosacea is mediated by dysregulated immune response. However, increasing evidence shows that systemic and local oxidative stress may be associated with disease activity in relation to the severity of symptoms caused by MGD [99]. Increased expression of toll-like receptor 4 has been associated with oxidative stress and disease severity in ocular rosacea [100].

#### Treatment challenges and IPL evidence in ocular rosacea

Conventional pharmacological treatments for ocular rosacea are limited by the incomplete control of abnormal lid-margin vasculature, recurrence of inflammation, and potential adverse effects of long-term systemic therapy [5, 64, 96, 101, 102]. Since IPL can target abnormal periocular vessels and may reduce lid-margin inflammation, it has been explored as part of multidisciplinary management in selected patients, especially those with concomitant telangiectasia, inflammation, and MGD [6, 64, 103]. Existing studies have suggested improvements in tear film stability, symptom scores, lid-margin vascular signs, and inflammatory or microbiome-related parameters on the ocular surface after IPL treatment combined with MGX [6, 22, 50, 64, 103]. However, the current evidence is still more limited than that for MGD-related evaporative dry eye. Controlled studies are needed to determine the independent contribution of IPL, optimal protocol, and long-term efficacy [6, 64].

### Chalazion

#### Disease essence and pathogenesis

Chalazion is a chronic, sterile, lipid-granulomatous inflammation caused by the obstruction of the meibomian gland duct [11, 79]. It is often associated with MGD and an imbalance in the eyelid microenvironment [104]. Research has shown that Demodex mite infestation may be an important environmental trigger, particularly in children. Prospective studies reported detection rates as high as 52.9%. Mite-associated glandular obstruction and the subsequent chronic inflammation may contribute to disease recurrence [104]. Chalazion not only represents a common eyelid mass but is also a specific manifestation of an imbalance in the periocular microenvironment [7, 66, 105, 106].

#### Management challenges and IPL evidence in recurrent or MGD-associated chalazion

Conventional management of chalazion includes warm compresses, topical treatment, corticosteroid injection, and incision and curettage [66, 79, 106, 107]. However, these approaches may be limited by recurrence, discomfort, scarring, or the need for anesthesia in pediatric patients [7, 66, 105, 108]. Therefore, IPL has been explored as a non-invasive adjunctive or alternative option in selected recurrent or MGD-associated chalazion cases [7, 65, 73, 95, 104, 108, 109]. Existing studies suggest that IPL may promote lesion resolution and reduce recurrence, possibly by improving meibomian gland function and eyelid microenvironment. However, most available studies are limited by small sample sizes, retrospective designs, or short follow-up periods. Larger controlled studies are needed before IPL can be recommended as a standard alternative to established treatments [7, 65, 73, 95, 108, 109]. Some studies have reported favorable outcomes in pediatric chalazion, recurrent adult chalazion, and IPL combined with MGX; however, these findings require confirmation in larger prospective studies [65, 73, 95, 108, 109].

An overview of the available clinical evidence is provided in Supplementary Table 1. The current evidence for IPL is the strongest in MGD-related evaporative dry eye, whereas evidence for blepharitis, ocular rosacea, and chalazion remains preliminary and phenotype-dependent. Allergic conjunctivitis is discussed separately as an emerging adjunctive application because the available evidence remains limited. Because of the substantial heterogeneity in study design, IPL parameters, adjunctive therapies, outcome measures, and follow-up duration, direct comparisons across studies remain limited, and the relative efficacy of IPL across different OSD phenotypes should therefore be interpreted with caution. Overall, these findings support IPL as a mechanism-oriented adjunctive therapy for selected refractory phenotypes rather than as a universal treatment for all OSDs. Therefore, treatment protocols should be stratified according to disease category, dominant pathology, and clinical features.

## Discussion

### Shift in OSD management philosophy

The use of IPL in ophthalmology reflects a shift toward mechanism-oriented adjunctive therapy for selected OSDs, particularly those characterized by MGD, lid-margin vascular abnormalities, Demodex-associated inflammation, or overlapping inflammatory mechanisms [6, 110]. Rather than serving as a universal treatment for OSDs, IPL may be most appropriately understood as a phenotype-directed intervention that targets specific pathological components, including meibomian gland obstruction, abnormal periocular vasculature, microbial or parasitic burden, and local inflammation [22]. This mechanistic rationale is the strongest in MGD-related evaporative dry eye, but its extension to other conditions requires more cautious interpretation. In clinical practice, IPL may offer a noninvasive, office-based adjunctive option for selected refractory patients. However, its role should be defined according to the disease subtype, baseline glandular structure, inflammatory status, and quality of available evidence.

### “Symptom–sign–structure dissociation”

“Symptom–sign–structure dissociation” is observed in relation to IPL therapy. Presumably, this phenomenon prioritizes the adjustment of cellular bioenergetic metabolism after IPL therapy, which is characterized by significantly increased ATP production. This increased ATP production may take priority over stabilizing the corneal nerve terminals and reducing neural hypersensitivity, rather than the immediate repair of the structural aspects of the glands. The levels of important pro-inflammatory cytokines, such as TNF-α and IL-6, are rapidly decreased, thereby optimizing the microenvironment. These mechanisms may partly explain why symptom relief precedes the resolution of clinical signs [50]. In addition to physical glandular decompression, this therapy may also influence tissue repair-related processes through neuroimmune modulation; however, this interpretation remains speculative. This contrasts markedly with conventional thermal therapies that are purely dedicated to physical unblocking.

### Application in pediatric patients

Although allergic conjunctivitis is primarily driven by immune-mediated mechanisms, including IgE-mediated hypersensitivity, ocular surface barrier dysfunction, and neurogenic inflammation, some refractory cases may present with lid-margin vascular dilation, inflammatory activation, and secondary MGD [9, 12, 58, 87]. Consequently, IPL has been explored as an adjunctive treatment because of its potential vascular photocoagulation, anti-inflammatory effects, and photobiomodulatory mechanisms. In pediatric patients, IPL may reduce reliance on prolonged corticosteroid use in selected refractory cases; however, its safety and efficacy require careful evaluation because multiple office-based sessions are still needed and the available evidence remains limited [105, 106]. A recent randomized controlled trial suggested that IPL combined with conventional anti-allergic therapy may improve the symptoms and signs of refractory seasonal allergic conjunctivitis in children [57]. Nevertheless, the evidence for IPL’s effectiveness in allergic conjunctivitis remains preliminary. Currently, its use should be considered investigational or adjunctive, particularly because allergic conjunctivitis only partially overlaps mechanistically with MGD-related diseases. Larger prospective randomized studies with longer follow-up periods are needed to clarify the treatment efficacy, safety, and appropriate patient selection.

### Limitations of current evidence

Although IPL has shown promising results, the evidence remains heterogeneous [3, 14, 45, 55, 111]. As shown in Supplementary Table 1, studies differed substantially in IPL device, filter, fluence, pulse mode, treatment interval, number of sessions, adjunctive therapy, outcome measures, and follow-up duration [24, 67, 68, 88, 112–114]. These variations limit direct comparisons across studies, complicate the interpretation of relative efficacy across disease phenotypes, and make it difficult to define standardized treatment protocols. In addition, long-term maintenance schedules remain insufficiently defined, and the durability of IPL benefits as well as the optimal timing and frequency of maintenance treatment require further investigation before broader clinical adoption [3, 45, 55, 111]. In addition, follow-up is often limited to weeks or months, with only a few studies assessing long-term outcomes; therefore, the durability of IPL benefits and the need for maintenance therapy remain unclear [4, 52, 75, 88, 115, 116]. Practical factors, including cost, device availability, operator experience, and repeated in-office visits may also limit real-world implementation [3, 55, 111]. Safety should also be evaluated using standardized protocols, particularly with respect to skin phototype, upper eyelid treatment, ocular protection, and device/operator variability [24, 67–70, 112–114]. Future studies should standardize the treatment parameters, adverse-event reporting, maintenance schedules, and follow-up outcomes [3, 14, 45, 55, 111].

### Future research directions

Although IPL therapy has shown promising results, especially in MGD-related evaporative dry eye, its broader adoption across other OSDs requires stronger indication-specific evidence. Before incorporating IPL into standardized treatment algorithms beyond MGD-related dry eye, several key issues must be addressed. First, future studies should move beyond clinical outcomes and further clarify the cellular and molecular mechanisms by which IPL modulates the immune microenvironment of the ocular surface, including its potential effects on glandular repair pathways, mast-cell activity, and Th2-related inflammatory signaling. Such mechanistic studies may support the development of phenotype-stratified phototherapy, in which IPL protocols are individualized according to the dominant disease phenotype, such as vascular-inflammatory, glandular-obstructive, or neurometabolic patterns. For example, IPL may be particularly relevant for vascular-inflammatory phenotypes, whereas neurometabolic phenotypes may require comparative or combined studies of IPL and LLLT. Multicenter studies are also needed to optimize treatment protocols according to the disease phenotype. For instance, a modified “five-flash” IPL technique has been proposed to enhance efficacy in patients with MGD, thus demonstrating the potential for further clinical parameter optimization [32]. Current existing research has already shown that adjusting IPL treatment frequency may be a key factor in improving efficacy in moderate-to-severe disease rather than adjusting the energy fluence level [51, 54, 64, 79, 88, 117].

IPL responses are highly variable among patients. Several studies have shown that patients with advanced glandular structural damage, such as massive and continuous loss and thinning on the meibomian gland at baseline, respond poorly to IPL treatment, even when the marginal signs are mild [71]. Therefore, morphological evaluation of the glands should be considered in the clinical decision to predict treatment efficacy and personalize treatment.

Trends in combination energy treatment: as the limitations of monotherapy become apparent, “combined energy therapy” has emerged as a promising area of focus. Pilot studies suggest that IPL combined with multi-frequency radiofrequency treatment may produce additive or complementary effects, as IPL effectively reduces inflammation and improves vascular status, whereas radiofrequency provides long-term thermal energy outcomes that may support future investigations of tissue remodeling-oriented treatment strategies [28].

Biomarker-based precision therapy: to advance from the current trial-and-error treatment approach, intensive studies are needed to investigate the relevance of tear film biomarkers, such as changes in the levels of specific cytokines or neurotrophic factors. This will pave the way for the development of a treatment efficacy prediction system that aims to establish personalized, precision medicine as a treatment approach [23, 118]. Additionally, this will facilitate the development of health-economic evaluations and enable the inclusion of IPL therapy under medical insurance for more comprehensive medical treatments. It will then be transformed from the present “limited-access intervention” status to a more “accessible treatment option” [14]. Moreover, IPL use in the treatment of neurotrophic keratitis may also be extended [52, 95].

## Conclusion

Overall, IPL therapy represents a promising physical treatment strategy for refractory OSDs with overlapping inflammatory, vascular, microbial, neuroimmune, and meibomian gland abnormalities. Its clinical value may depend on matching the dominant disease phenotype with the most relevant IPL mechanisms, such as vascular modulation in ocular rosacea, meibum liquefaction in MGD, or the reduction of Demodex-associated inflammation in blepharitis. IPL may also help explain the symptom–sign dissociation observed in some patients, possibly through neuroimmune modulation before measurable structural recovery occurs. Its noninvasive, office-based nature offers a practical alternative for pediatric and treatment-resistant patients where long-term corticosteroid use or complex home care is difficult; however, IPL requires a structured course of repeated office-based sessions, which may reduce reliance on daily home-based treatment for some selected patients. Therefore, IPL must advance from an emerging procedure to a better-standardized and more phenotype-directed therapeutic option. Future research integrating combined energy strategies, phenotype-stratified phototherapy, and biomarker-driven personalization may improve the alignment of treatment mechanisms and disease phenotypes.

## Supplementary Information

Suppplementary Table 1

## Data Availability

The data used in this analysis were obtained from studies identified through a search of the PubMed database (https://pubmed.ncbi.nlm.nih.gov).

## Abbreviations

ATP: Adenosine triphosphate
IPL: Intense pulsed light
LLLT: low-level light therapy
MGD: Meibomian gland dysfunction
MGX: Meibomian gland expression
OSD: Ocular surface disease
OSDI: Ocular surface disease index
TBUT: Tear film breakup time
TNF-α: Tumour necrosis factor-α
MMP-9: Matrix metalloproteinase-9
IgE: Immunoglobulin E
